# BVR-A Deficiency Leads to Autophagy Impairment through the Dysregulation of AMPK/mTOR Axis in the Brain—Implications for Neurodegeneration

**DOI:** 10.3390/antiox9080671

**Published:** 2020-07-27

**Authors:** Chiara Lanzillotta, Ilaria Zuliani, Chirag Vasavda, Solomon H. Snyder, Bindu D. Paul, Marzia Perluigi, Fabio Di Domenico, Eugenio Barone

**Affiliations:** 1 Department of Biochemical Sciences “A. Rossi-Fanelli”, Sapienza University of Rome, 00185 Rome, Italy; chiara.lanzillotta@uniroma1.it (C.L.); ilaria.zuliani@uniroma1.it (I.Z.); marzia.perluigi@uniroma1.it (M.P.); 2 The Solomon H. Snyder Department of Neuroscience, Johns Hopkins University School of Medicine, Baltimore, MD 21205, USA; cvasavda@jhmi.edu (C.V.); ssnyder@jhmi.edu (S.H.S.); bpaul8@jhmi.edu (B.D.P.); 3 Department of Psychiatry and Behavioral Sciences, Johns Hopkins University School of Medicine, Baltimore, MD 21205, USA; 4 Department of Pharmacology and Molecular Sciences, Johns Hopkins University School of Medicine, Baltimore, MD 21205, USA

**Keywords:** AMPK, autophagy, biliverdin reductase, mTOR, neurodegeneration, oxidative stress

## Abstract

Biliverdin reductase-A (BVR-A) impairment is associated with increased accumulation of oxidatively-damaged proteins along with the impairment of autophagy in the brain during neurodegenerative disorders. Reduced autophagy inhibits the clearance of misfolded proteins, which then form neurotoxic aggregates promoting neuronal death. The aim of our study was to clarify the role for BVR-A in the regulation of the mTOR/autophagy axis by evaluating age-associated changes (2, 6 and 11 months) in cerebral cortex samples collected from BVR-A knock-out (BVR-A^−/−^) and wild-type (WT) mice. Our results show that BVR-A deficiency leads to the accumulation of oxidatively-damaged proteins along with mTOR hyper-activation in the cortex. This process starts in juvenile mice and persists with aging. mTOR hyper-activation is associated with the impairment of autophagy as highlighted by reduced levels of Beclin-1, LC3β, LC3II/I ratio, Atg5–Atg12 complex and Atg7 in the cortex of BVR-A^−/−^ mice. Furthermore, we have identified the dysregulation of AMP-activated protein kinase (AMPK) as a critical event driving mTOR hyper-activation in the absence of BVR-A. Overall, our results suggest that BVR-A is a new player in the regulation of autophagy, which may be targeted to arrive at novel therapeutics for diseases involving impaired autophagy.

## 1. Introduction

Biliverdin reductase (BVR) is an evolutionarily conserved and ubiquitously expressed enzyme involved in the heme degradation pathway. During its catabolism, heme is initially converted by heme oxygenase (HO) to biliverdin, which is then reduced to bilirubin through the action of BVR [1,2]. Two different isoforms of BVR were detected in humans and named BVR-A and BVR-B [3]. Although both isozymes catalyze the reduction of biliverdin, BVR-A selectively reduces biliverdin IXα to bilirubin IXα, one of the strongest endogenous antioxidants [3,4]. For that reason, BVR-A has been studied for a long time as an antioxidant enzyme capable of counteracting oxidative stress-induced alterations [1,2,5].

Notwithstanding, BVR-A is a protein with pleiotropic functions [1,2]. BVR-A can translocate to the nucleus acting as a bZIP-type transcription factor, controlling the expression of genes involved in stress-responses [6,7,8,9,10]. Furthermore, BVR-A was reported to play a pivotal role in regulating inflammatory responses [3,10]. Interestingly, several sequence motifs within the BVR-A structure were identified as possible protein–protein interaction sites [11]. BVR-A was shown to be a Ser/Thr/Tyr kinase able to regulate a number of pathways involved in cell growth, differentiation and survival [1,3,12]. In particular, BVR-A was identified as a novel regulator of the insulin/IGF1 signaling in vitro [13,14,15] and recent studies showing that loss of BVR-A impairs insulin signaling activation and cell metabolism also in vivo support this hypothesis [16,17,18,19].

In addition to its role in the periphery, it is becoming increasingly clear that BVR-A plays important roles in the central nervous system. Mice lacking BVR-A and thus bilirubin, are particularly vulnerable to the toxic effects of superoxide (O_2_^•−^), which promotes excitotoxicity and neuronal cell death [20]. Neuroblastoma cells lacking BVR-A are unable to activate the Akt/GSK3β axis, thus appearing more susceptible to H_2_O_2_-induced oxidative damage [21]. Furthermore, induction of BVR-A was reported to prevent hippocampal cell death by inhibiting DNA fragmentation and generation of reactive oxygen species in an ischemic model [22].

In parallel, studies from our group highlighted that increased oxidative stress levels lead to BVR-A oxidative damage in the brain thus favoring neuronal dysfunctions both during ageing process [9,12,23], neurodegenerative diseases (i.e., mild cognitive impairment (MCI) and Alzheimer’s disease (AD)) [12,19,21,23,24,25,26,27] and genetic disorders such as Down Syndrome (DS) [28]. Then, as in a vicious cycle, the impairment of BVR-A favors the accumulation of AD neuropathological markers, that is, amyloid beta (Aβ), hyper-phosphorylated tau and increased oxidative stress-induced damage to proteins and lipids [12,19,21,23].

One of the main clearance systems through which cells recycle nutrients, discard damaged components and promote adaptation to stress conditions is autophagy [29,30,31]. An increasing number of studies supported a role for aberrant autophagy in the accumulation of protein aggregates observed in the brain during aging or neurodegenerative disorders [32,33]. The mammalian target of rapamycin (mTOR) is the master regulator of autophagy [34,35] and an hyper-activation of mTOR—which negatively regulates autophagy induction—along with the reduction of the autophagic flux was reported in AD [36,37,38,39]. Furthermore, the reduction of autophagic flux has also been implicated in the accumulation of oxidatively modified proteins during aging and neurodegenerative disorders [36,40,41,42,43,44], thus exacerbating the increase of oxidative/nitrosative damage in the brain (reviewed in Reference [45]). Conversely, inducing autophagy through rapamycin-mediated inhibition of mTOR appears to be beneficial in slowing down the neurodegenerative process [38,46,47].

In light of our previous data showing that reduced BVR-A levels or impaired BVR-A activity parallels the hyper-activation of mTOR in both human brain tissue [21,27,36] and in animal models of AD [12,19] or aging [23], the main goal of this study was to evaluate age-associated alterations of mTOR and autophagic flux in the cerebral cortex of a knock-out mouse model for BVR-A (BVR-A^−/−^). We provide evidence that loss of BVR-A promotes mTOR hyper-activation, leading to the impairment of autophagy and the subsequent accumulation of oxidatively-damaged proteins.

## 2. Material and Methods

### 2.1. Animals

Cerebral cortex samples from 2, 6 and 11 months-old BVR^−/−^ and C57BL/6 j mice (*n* = 4/group, all males) were kindly provided by Dr. Paul BD and Prof. Snyder SH. BVR-A^−/−^ mice were generated as previously described [20]. Briefly, BVR^−/−^ mice were generated at Ozgene (Australia) on a C57BL/6J background. To ensure the loss of bilirubin biosynthesis, exon 3 of BLVRA was flanked by two LoxP sequences for deletion via the Cre recombinase. BVR^−/−^ mice were then backcrossed to wild-type C57BL/6J mice for four or more generations before these studies. Age-matched wild-type C57BL/6J mice were used as controls. All experiments were performed in accordance with protocol (ID #MO18M409) approved by the Animal Care and Use Committee (ACUC) at the Johns Hopkins University School of Medicine.

### 2.2. Samples Preparation

Total protein extracts were prepared in RIPA buffer (pH = 7.4) containing 50 mM Tris-HCl (pH = 8), 150 mM NaCl, 1% NP-40, 0.5% sodium deoxycholate,1 mM EDTA, 0.1% SDS, together with phosphatase and protease inhibitor (539132, Millipore, 1:100; P0044; Sigma-Aldrich, St. Louis, MO, USA; 1:100). Samples were sonicated on ice and then centrifuged at 16,000× *g* rpm at 4 °C for 30 min to remove cellular debris. Supernatants were collected to determine total protein concentrations by the BCA method (Pierce, Rockford, IL, USA).

### 2.3. Slot Blot Analysis

To evaluate total protein-bound (i) 4-hydroxy-2-nonenal (HNE) and (ii) 3-nitrotyrosine (3-NT) levels, 3 μL of cortical proteins homogenate were incubated with 6 μL of Laemmli Buffer (0.125 M Tris base pH = 6.8, 4% (*v*/*v*) SDS and 20% (*v*/*v*) glycerol) for 20 min at room temperature and then loaded onto nitrocellulose membrane as described below. Proteins (250 ng) were loaded in each well on a nitrocellulose membrane under vacuum using a slot blot apparatus. Membranes were blocked for 1 h at room temperature with 3% of bovine serum albumin (SERVA Electrophoresis GmbH, Heidelberg, Germany) in Tris-buffered saline (TBS) solution containing 0.01% Tween 20 and incubated at room temperature for 2 h with the following primary antibodies: anti HNE polyclonal antibody (1:2000, Novus Biologicals, Abingdon, UK, # NB100-63093) or an anti 3-NT polyclonal antibody (1:1000, Sigma-Aldrich, St Louis, MO, USA, # N5538). Then, membranes were washed three times with TBS solution containing 0.01% Tween 20 and incubated for 1 h at room temperature with the respective alkaline phosphatase secondary antibodies from Sigma-Aldrich: anti-mouse (1:3000, Sigma-Aldrich, St Louis, MO, USA, A1293;) for 3-NT primary antibody and anti-goat (1:3000, Sigma-Aldrich, St Louis, MO, USA, A4187) for HNE primary antibody. The membranes were later washed three times in TBS solution containing 0.01% Tween 20 and developed with Sigma Fast BCIP/NBT (5-Bromo-4-chloro-3-indolyl phosphate/Nitro blue tetrazolium substrate). Blots were dried, acquired with Chemi-Doc MP imaging system Bio-Rad Laboratories, # 17001402) (Bio-Rad, Hercules, CA, USA) and analyzed using Image Lab 6.0 software (Bio-Rad, Hercules, CA, USA).

No non-specific binding of antibody to the membrane was observed.

### 2.4. Western Blot Analysis

For Western blot, 10 μg of protein were separated by 4–15% gradient sodium dodecyl sulfate–polyacrylamide gel electrophoresis (SDS-PAGE), using Criterion TGX (Tris-Glycine extended) Stain-Free precast gels (Bio-Rad, Hercules, CA, USA) in Tris/Glycine/SDS (TGS) Running Buffer (Bio-Rad Laboratories, # 1610772). All the samples were loaded on the same gel by using 26-well gel (Bio-Rad Laboratories, # 5678085). Immediately after electrophoresis, the gel was then placed on a Chemi/UV/Stain-Free tray and then placed into a ChemiDoc MP imaging System (Bio-Rad Laboratories, # 17001402) and UV-activated based on the appropriate settings with Image Lab Software (Bio-Rad Laboratories) to collect total protein load image. Following electrophoresis and gel imaging, the proteins were transferred via the TransBlot Turbo semi-dry blotting apparatus (Bio- Rad Laboratories, # 1704150) onto nitrocellulose membranes (Bio-Rad, Hercules, CA, USA, # 162–0115). Membranes were blocked with 3% of bovine serum albumin (SERVA Electrophoresis GmbH, Heidelberg, Germany) in TBS solution containing 0.01% Tween 20 and incubated over night at 4 °C with the following primary antibodies: AMPK (1:500, Santa Cruz, Santa Cruz, CA, USA, # sc-774461), p^Thr173^AMPK (1:1000; Cell Signaling, Bioconcept, Allschwill, Switzerland, # 2535), Atg7 (1:1000, Santa Cruz, Santa Cruz, CA, USA; sc-376212), Atg5 (1:1000, Santa Cruz, Santa Cruz, CA, USA, sc-133158), Beclin-1 (1:1000; Cell Signaling, Bioconcept, Allschwill, Switzerland, # 3738s), BVR (1:5000, Abcam, Cambridge, UK, AB-90491), LAMP1 (1:1000, Santa Cruz, Santa Cruz, CA, USA; sc-20011), LC3β (1:1000, Santa Cruz, Santa Cruz, CA, USA; sc-16755), LC3 (1:1000, Novus Biotechnology, Centennial, CO, USA, NB1002220), mTOR (1:1000; Cell Signaling, Bioconcept, Allschwill, Switzerland # 2983s), p^Ser2448^mTOR (1:1000; Cell Signaling, Bioconcept, Allschwill, Switzerland, # 5536s), SQSTM1 (1:1000, Santa Cruz, Santa Cruz, CA, USA sc-28359).

Subsequently, membranes were incubated at room temperature with the respective horseradish peroxidase-conjugated secondary antibodies for 1 h: anti-rabbit (1:20,000, Bio-Rad, Hercules, CA, USA, L005661), anti-mouse (1:20000, Bio-Rad, Hercules, CA, USA, L005662) or anti-goat IgG (1:3000, Sigma-Aldrich, St Louis, MO, USA, A5420). At the end, membranes were developed with Clarity enhanced chemiluminescence (ECL) substrate (Bio-Rad, Hercules, CA, USA, # 1705061) and then acquired with Chemi-Doc MP (Bio-Rad, Hercules, CA, USA). Determination of relative abundance of each protein was calculated using Image Lab 6.0 software (Bio-rad Laboratories, Hercules, CA, USA).

### 2.5. Statistical Analysis

Statistical analyses were performed using 2-way ANOVA analysis using Fisher’s LSD test. Data are expressed as mean ± SEM per group. All statistical analyses were performed using Graph Pad Prism 8.0 software (GraphPad, La Jolla, CA, USA).

## 3. Results

### 3.1. Oxidatively-Damaged Proteins Accumulation along with mTOR Hyper-Activation Occur in BVR-A^−/−^ Mice

Age-associated changes to levels of protein-bound HNE and 3-NT and mTOR expression and activation were evaluated in the cortex of BVR-A^−/−^ and WT mice at 2, 6 and 11 months of age, with the aim to understand whether loss of BVR-A influences these processes. To highlight both genotype- and age-associated effects, the results throughout the paper are presented by using two different graphs (plotted with the same results). In the first graph (columns) we highlighted differences observed among groups (WT vs. BVR-A^−/−^), while in the second graph (dots) we highlighted changes with aging for each group.

**Figure 1:**
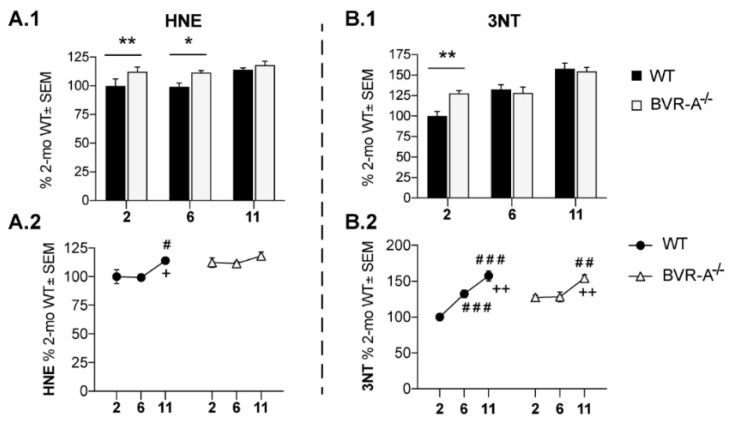
Increased oxidatively-damaged proteins accumulation in the cerebral cortex of Biliverdin reductase-A (BVR-A^−/−^) mice. Slot Blot quantification of 4-hydroxy-2-nonenal (HNE) (**A.1**,**A.2**) and 3-NT levels (**B.1**,**B.2**) evaluated in the cerebral cortex of wild-type (WT) and BVR-A^−/−^ mice at 2 (*n* = 4), 6 (*n* = 4) and 11 (*n* = 4) months of age. Data are expressed as percentage of WT mice at 2 months set as 100%. Columns were used to show differences among the groups (WT vs. BVR-A^−/−^) while dots to show age-associated changes within each group. Data are expressed as Mean ± SEM. For columns: * *p* < 0.05, ** *p* < 0.01 vs. WT (2-way ANOVA with Fisher’s LSD test). For dots: # *p* < 0.05, ## *p* < 0.01, ### *p* < 0.001 vs. 2 months; + *p* < 0.05, ++ *p* < 0.01 vs. 6 months (2-way ANOVA with Fisher’s LSD test).

Our data demonstrate an increase of protein-bound HNE adducts both at 2 (+15%, *p* < 0.05) and 6 months of age (+12%, *p* < 0.05) in the cortex of BVR-A^−/−^ with respect to WT mice (Figure 1A.1,A.2). Similarly, increased 3-NT levels in BVR-A^−/−^ mice at 2 months (+27%, *p* < 0.01) were observed (Figure 1B.1,B.2). By evaluating HNE and 3-NT data with respect to age progression (Figure 1B,D) a different effect for aging is evident for WT and BVR-A^−/−^ mice. Indeed, while an increase of HNE and 3-NT levels occurs in WT mice only at an adult/old age, BVR-A^−/−^ mice show elevated markers of proteins oxidation starting at 2 months (Figure 1A.2,B.2). Accordingly, 2-way ANOVA analysis supports the effect of age for both HNE and 3-NT changes (F (2, 18) = 5.18, *p* < 0.05 and F (2, 18) = 28.70, *p* < 0.0001, respectively) and age × genotype interaction for 3-NT (F (2, 18) = 5.14, *p* < 0.05) (Table 1). These data suggest that BVR-A might play a crucial role in the protection from oxidatively-damaged proteins accumulation, in agreement with [20].

**Figure 2:**
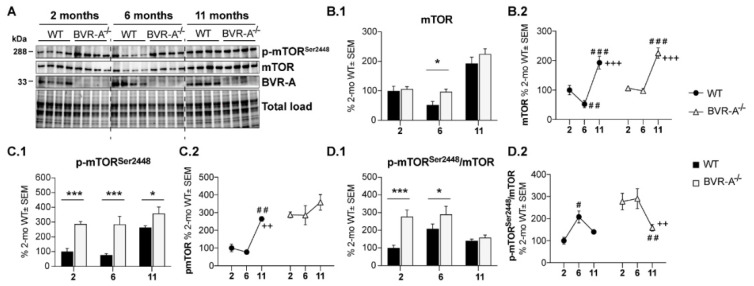
mTOR hyper-activation occurs in the cerebral cortex of BVR-A^−/−^ mice. Representative western blot images (**A**) and densitometric evaluation of mTOR (**B.1**,**B.2**), p-mTOR^Ser2448^ (**C.1**,**C.2**) and p-mTOR^Ser2448^/mTOR ratio (**D.1**,**D.2**) in the cerebral cortex of WT and BVR-A^−/−^ mice at 2 (*n* = 4), 6 (*n* = 4) and 11 (*n* = 4) months of age. Protein levels were normalized per total protein load. Data are expressed as percentage of WT mice at 2 months set as 100%. Columns were used to show differences among the groups (WT vs. BVR-A^−/−^) while dots to show age-associated changes within each group. Data are expressed as Mean ± SEM. For columns: * *p* < 0.05, *** *p* < 0.001 vs. WT (2-way ANOVA with Fisher’s LSD test). For dots: # *p* < 0.05, ## *p* < 0.01, ### *p*< 0.001 vs. 2 months; ++ *p* < 0.01, +++ *p* < 0.001 vs. 6 months (2-way ANOVA with Fisher’s LSD test).

To determine whether the early increase of oxidatively-damaged proteins in BVR-A^−/−^ mice is associated with the mTOR/autophagy axis impairment, we evaluated the activation state of mTOR complex. In fact, mTOR is a core component of the multiprotein complex mTORC1, through which it acts to inhibit autophagic induction [48]. Sustained mTOR activation (indexed by Ser2448 phosphorylation) is well-known to mediate the drop of autophagy function by interacting with components of the autophagosome formation process [34,35]. In our experimental setting, mTOR protein levels did not show any significant differences between WT and BVR-A^−/−^ mice except for a significant increase observed at 6 months in BVR-A^−/−^ mice with respect to WT (+45%, *p* < 0.05) (Figure 2B.1). Significant changes for mTOR protein levels with aging are evident in both the two groups (Figure 2B.2). When we evaluated the active form (p-mTOR^Ser2448^), we found significantly elevated levels in BVR-A^−/−^ with respect to WT mice for each age considered for the study (+187%, *p* < 0.001 at 2 months, +208%, *p* < 0.001 at 6 months and +94%, *p* < 0.05 at 11 months) (Figure 2C.1). As observed for protein oxidation, mTOR phosphorylation levels increase early in life in BVR-A^−/−^ mice with respect to WT (Figure 2C.1,C.2) suggesting a differential aging process between the two groups of comparison. Finally, we analyzed the active form per total protein levels ratio (p-mTOR^Ser2448^/mTOR) as an index of mTOR activation process in each subgroup. Our results show that p-mTOR^Ser2448^/mTOR ratio is significantly increased in BVR-A^−/−^ mice with respect to WT both at 2 (+177%, *p* < 0.001) and 6 months (+83%, *p* < 0.05) (Figure 2D.1). In addition, age-associated changes of p-mTOR^Ser2448^/mTOR ratio progress with a different profile in BVR-A^−/−^ compared WT mice (Figure 2D.2). Consistently with the above data, 2-way ANOVA analysis demonstrates that both age and genotype largely influence changes of mTOR protein levels (age: F (2, 18) = 45.89, *p* < 0.0001; genotype: F (1, 18) = 5.33 *p* < 0.05) and mTOR phosphorylation (age: F (2, 18) = 10.53, *p* < 0.001; genotype: F (1, 18) = 40.78, *p* < 0.0001) (Table 1). Together, these observations suggest that loss of BVR-A promotes the hyper-activation of mTOR.

### 3.2. mTOR Hyper-Active Leads to Impaired Autophagy in BVR-A^−/−^ Mice

To understand whether the hyper-activation of mTOR leads to impaired autophagy in BVR-A^−/−^ mice, we focused on the analysis of proteins crucially involved in the process of autophagosome nucleation/formation/maturation.

**Figure 3:**
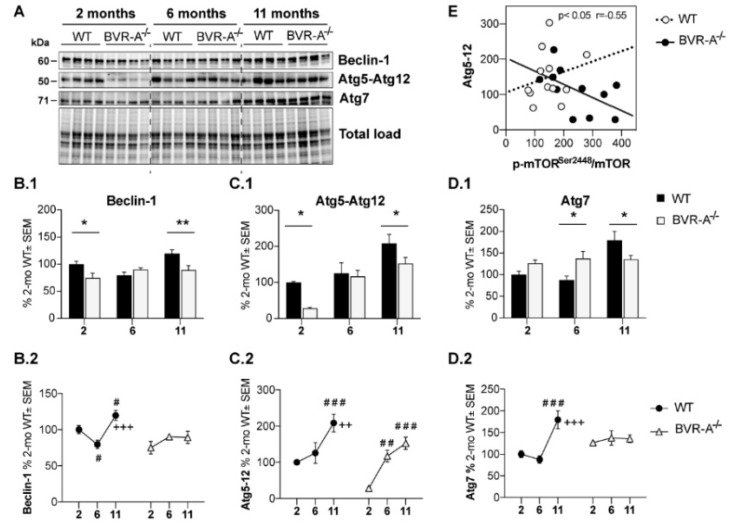
Reduced autophagosome nucleation occurs in the cerebral cortex of BVR-A^−/−^ mice. Representative western blot images (**A**) and densitometric evaluation of Beclin-1(**B.1**,**B.2**), Atg5–Atg12 complex (**C.1**,**C.2**) and Atg7 (**D.1**,**D.2**) levels in the cerebral cortex of WT and BVR-A^−/−^ mice at 2 (*n* = 4), 6 (*n* = 4) and 11 (*n* = 4) months of age. Protein levels were normalized per total protein load. Data are expressed as percentage of WT mice at 2 months set as 100%. Columns were used to show differences among the groups (WT vs. BVR-A^−/−^) while dots to show age-associated changes within each group. Data are expressed as Mean ± SEM. For columns: * *p* < 0.05, ** *p* < 0.01 vs. WT (2-way ANOVA with Fisher’s LSD test). For dots: # *p* < 0.05, ### *p* < 0.001 vs. 2 months; ++ *p* < 0.01, +++ *p* < 0.001 vs. 6 months (2-way ANOVA with Fisher’s LSD test). (**E**) Pearson correlation between Atg5–Atg12 and p-mTOR^Ser2448^/mTOR ratio in WT and BVR-A^−/−^ mice.

The autophagosome nucleation is driven by the Beclin/parkin/PI3K class III complex [33]. Variations of Beclin-1 thus reflect alterations in the nucleation step and is a well-accepted marker of impairment of the process. Our results show a significant reduction of Beclin-1 protein levels in BVR-A^−/−^ mice both at 2 (−25%, *p* < 0.05) and 11 months (30%, *p* < 0.01) with respect to WT mice (Figure 3B.1). Furthermore, age-associated changes of Beclin-1 demonstrate a different profile between WT and BVR-A^−/−^ mice (Figure 3B.2). Consistently, 2-way ANOVA analysis reveals the influence of age (F (2, 18) = 5.13, *p* < 0.05), genotype (F (1, 18) = 4.6, *p* < 0.05) and age x genotype interaction (F (2, 18) = 5.59, *p* < 0.05) with regard to changes of Beclin-1 protein levels (Table 1).

To further explore whether changes of autophagosome nucleation are followed by the alteration of components of the autophagosome formation, lysosomal cargo gathering and trafficking, we examined the expression levels of autophagy-related proteins (Atg) [49]. Alterations of proteins involved in these steps can compromise the clearance of damaged organelles and aggregated proteins and can trigger the increase of oxidative damage [50,51]. Similar to Beclin-1, a significant decrease of Atg5–Atg12 complex levels both at 2 (−72%, *p* < 0.05) and 11 months (−56%, *p* < 0.05) in BVR-A^−/−^ with respect to WT mice was observed (Figure 3C.1). Furthermore, while the levels of Atg5–Atg12 complex increase with age both in WT and BVR-A^−/−^ (Figure 3C.2), the profile is different thus underling a defective autophagosome maturation in mice lacking BVR-A. In particular, Atg5–Atg12 complex levels are reduced at 2 months in BVR-A^−/−^ mice, after which they consistently rise at 6 months and at 11 months. However, Atg5–Atg12 complex levels do not reach similar levels in BVR-A^−/−^ mice as in WT mice (Figure 3C.2). Changes of Atg5–Atg12 complex levels are mainly affected by age (F (2, 18) = 2.30, *p* < 0.0001) and genotype (F (1, 18) = 9.27, *p* < 0.01) (Table 1). In addition, we observed that Atg7 protein levels were significantly increased at 6 months (+50%, *p* < 0.05) but they are significantly reduced at 11 months (−44%, *p* < 0.05) in BVR-A^−/−^ with respect to WT mice (Figure 3D.1). Also, age-associated changes for Atg7 occur in a different manner in WT and BVR-A^−/−^ mice (Figure 3D.2), thus strengthen the hypothesis that Atg7 activity is compromised in the absence of BVR-A. Indeed, 2-way ANOVA analysis reveals that changes for Atg7 protein levels are mainly affected by age (F (2, 18) = 8.26, *p* < 0.01) and age × genotype interaction (F (2, 18) = 7.34, *p* < 0.01) (Table 1).

Thus, observed alterations for Atg5–Atg12 and Atg7 proteins suggest that a disruption in the elongation phase of the autophagosome formation and thus an impairment of autophagy occur in BVR-A^−/−^ mice. Indeed, when exploring for possible associations of altered Atg5–Atg12 in the overall groups, we observed that lower Atg5–Atg12 levels were associated with higher p-mTOR^Ser2448^/mTOR ratio (*p* < 0.05, *r* = −0.55) in BVR-A^−/−^ but not in WT mice, suggesting that mTOR hyper-activation observed in mice lacking BVR-A could be responsible for the defects in the elongation phase of the autophagosome (Figure 3E).

**Figure 4:**
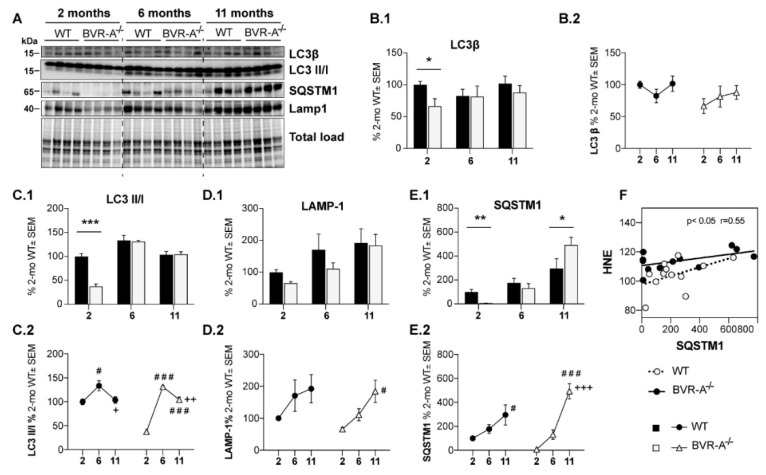
Reduced autophagosome maturation occurs in the cerebral cortex of BVR-A^−/−^ mice. Representative western blot images (**A**) and densitometric evaluation of LC3β (**B.1**,**B.2**), LC3 II/I ratio (**C.1**,**C.2**), LAMP-1 (**D.1**,**D.2**) and SQSTM1 (**E.1**,**E.2**) levels in the cerebral cortex of WT and BVR-A^−/−^mice at 2 (*n* = 4), 6 (*n* = 4) and 11 (*n* = 4) months of age. Protein levels were normalized per total protein load. Data are expressed as percentage of WT mice at 2 months set as 100%. Columns were used to show differences among the groups (WT vs. BVR-A^−/−^) while dots to show age-associated changes within each group. Data are expressed as Mean ± SEM. For columns: * *p* < 0.05, ** *p* < 0.01, *** *p* < 0.001 vs. WT (2-way ANOVA with Fisher’s LSD test). For dots: # *p* < 0.05, ### *p* < 0.001 vs. 2 months; + *p* < 0.05, ++ *p* < 0.01, +++ *p* < 0.001 vs. 6 months (2-way ANOVA with Fisher’s LSD test). (**F**) Pearson correlation between SQSTM1 and HNE levels in WT and BVR-A^−/−^ mice.

Subsequently, we analyzed changes of LC3β protein and of its cleaved forms as indexes of autophagosome maturation. LC3β is cleaved (by the removal of the C-terminal 22 amino acids) into the cytosolic form LC3-I, which is then lipidated and converted to LC3-II; this isoform is anchored in the inner and outer membrane of the autophagosome and gets released after autolysosomal degradation. Therefore, the evaluation of LC3II/I ratio is widely used as marker of autophagic flux [52,53]. Both total LC3β protein (−34%, *p* < 0.05) and the LC3II/I ratio (−63%, *p* < 0.0001) were significantly reduced in BVR-A^−/−^ with respect to WT mice at 2 months (Figure 4B.1,C.1). No significant changes with age were observed for total LC3β protein within each group (Figure 4B.2), while LC3II/I ratio seems to be more altered in BVR-A^−/−^ compared to WT (Figure 4C.2).

We further evaluated the lysosomal-associated membrane protein 1 (LAMP1), a marker of lysosome mass, that is essential in maintaining lysosomal integrity [54]. No significant changes were observed for LAMP1 protein levels between BVR-A^−/−^ and WT mice (Figure 4D.1). Rather, an increase of LAMP1 protein levels occurs with age in BVR-A^−/−^ mice (Figure 4D.2). Consistently, 2-way ANOVA analysis shows that changes of LAMP1 are mainly driven by age (F (2, 18) = 7.5, *p* < 0.01).

Finally, we analyzed SQSTM1 protein levels, a well-established marker of autophagic degradation efficiency. SQSTM1 is a multidomain protein that drives ubiquitinated substrates to lysosomal degradation during selective autophagy. Since SQSTM1 itself is degraded by autolysosomes, its measurement serves as an indirect evaluation of autophagic efficiency with its protein levels being inversely correlated with autophagic degradation [52]. SQSTM1 protein levels are strongly reduced at 2 months (−92%; *p* < 0.01) but consistently increased at 11 months (+246%, *p* < 0.05) in BVR-A^−/−^ mice compared to WT mice (Figure 4E.1). Changes within each group appear to progress with the same profile during aging, although a more pronounced elevation of SQSTM1 protein levels occur in BVR-A^−/−^ mice (Figure 4E.2). Indeed, 2-way ANOVA analysis reveals a major role for age (F (2, 18) = 25.27, *p* < 0.0001) and age x genotype interaction (F (2, 18) = 5.022, *p* < 0.001) Table 1). In addition, increased SQSTM1 protein levels are positively associated with HNE levels only in BVR-A^−/−^ but not in WT mice (Figure 4F), thus suggesting that degradation of damaged proteins through autophagy could be compromised in the absence of BVR-A.

Together, these results suggest that the absence of BVR-A leads to an impairment of the autophagosome formation.

### 3.3. Reduced AMPK Protein Levels and Activation Lead to mTOR Hyper-Activation in BVR-A^−/−^ Mice

To better understand the mechanism through which loss of BVR-A favors the aberrant regulation of the mTOR/autophagy axis in mouse brain, we estimated the involvement of AMP-activated protein kinase (AMPK), a key energy sensor that regulates cellular homeostasis [55], also by acting as an upstream inhibitor for mTOR [55].

**Figure 5:**
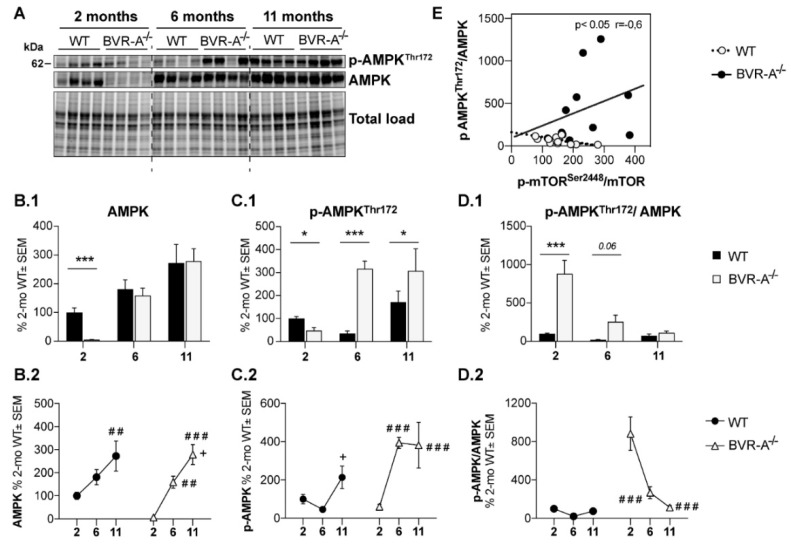
Reduced AMPK levels and activation in the cerebral cortex of BVR-A^−/−^ mice. Representative western blot images (**A**) and densitometric evaluation of AMPK (**B.1**,**B.2**), p-AMPK^Thr172^ (**C.1**,**C.2**) and p-AMPK^Thr172^/AMPK ratio (**D.1**,**D.2**) in the cerebral cortex of WT and BVR-A^−/−^ mice at 2 (*n* = 4), 6 (*n* = 4) and 11 (*n* = 4) months of age. Protein levels were normalized per total protein load. Data are expressed as percentage of WT mice at 2 months set as 100%. Columns were used to show differences among the groups (WT vs. BVR-A^−/−^) while dots to show age-associated changes within each group. Data are expressed as Mean ± SEM. For columns: * *p* < 0.05, *** *p* < 0.001 vs. WT (2-way ANOVA with Fisher’s LSD test). For dots: ## *p* < 0.01, ### *p* < 0.001 vs. 2 months; + *p* < 0.05 vs. 6 months (2-way ANOVA with Fisher’s LSD test). (**E**) Pearson correlation between p-AMPK^Thr172^/AMPK ratio and p-mTOR^Ser2448^/mTOR ratio in WT and BVR-A^−/−^ mice.

Changes of AMPK protein levels and phosphorylation at Thr172 (p-AMPK^Thr172^, active form) were evaluated. Our analysis shows that AMPK protein levels are drastically reduced at 2 months (−95%, *p* < 0.001) in BVR-A^−/−^ compared to WT mice (Figure 5B.1). The evaluation p-AMPK^Thr172^ levels shows a significant reduction at 2 months of age (−52%, *p* < 0.05) but a significant increase both at 6 (−281%, *p* < 0.01) and 11 months (−136%, *p* < 0.05) in BVR-A^−/−^ with respect to WT mice (Figure 5C.1). Finally, the analysis of AMPK active form per total protein levels ratio (p-AMPK^Thr172^/AMPK) as an index of AMPK activation process, reveals a strong increase at 2 months (+1188%, *p* < 0.0001) that is nearly significant at 6 months (+275%, *p* = 0.06) but is not significantly different at 11 months in BVR-A^−/−^ with respect to WT mice (Figure 5D.1).

Furthermore, age-associated changes of AMPK within each group suggest that AMPK is dysregulated in BVR-A^−/−^ mice. We found that AMPK protein levels increase with age in the cerebral cortex of BVR-A^−/−^ mice in a greater fashion with respect to WT mice (Figure 5B.2). Moreover, age-associated changes profiles for both p-AMPK^Thr172^ and p-AMPK^Thr172^/AMPK ratio are completely different when comparing WT and BVR-A^−/−^ mice. Age largely affects changes to AMPK protein levels (F (2, 18) = 18.45, *p* < 0.0001) (Table 1), while both age and genotype impact on changes of p-AMPK^Thr172^ (age: F (2, 18) = 7.42, *p* < 0.01; genotype: F (1, 18) = 11.55, *p* < 0.01; age x genotype: F (2, 18) = 5.74, *p* < 0.05) and p-AMPK^Thr172^/AMPK ratio (age: F (2, 18) = 19.88, *p* < 0.0001; genotype: F (1, 18) = 36.12, *p* < 0.0001; age × genotype: F (2, 18) = 17.40, *p* < 0.0001) (Table 1).

Intriguingly, AMPK (p-AMPK^Thr172^/AMPK) and mTOR (p-mTOR^Ser2448^/mTOR) activation are negatively associated in WT mice, that is, increased AMPK activation parallels reduced mTOR activation, in agreement with the regulatory role for AMPK [55]. Conversely, this association is lost in BVR-A^−/−^ mice (Figure 5E), suggesting that AMPK fails to regulate mTOR, which becomes hyper-active. Furthermore, we found that levels of p-mTOR^Ser2448^, the active form of the protein, are significantly and positively associated with the levels of most of the autophagy-related proteins we evaluated in the current study only in WT and not in BVR-A^−/−^ mice (Table 2). This observation is in agreement with the concept that mTOR promotes autophagy inhibition only when aberrantly activated [55], as observed in BVR-A^−/−^ mice.

Hence, these results are suggestive of the hypothesis that loss of BVR-A impairs the AMPK-mediated inhibition of mTOR thus leading to hyper-active mTOR and autophagy inhibition.

## 4. Discussion

A growing number of studies support a role for impaired autophagy in the development of neurodegenerative diseases, whereby the accumulation of oxidized/unfolded protein contributes to their pathogenesis [33,56]. Previous data from our group collected with regard to AD, DS or aging suggested that mTOR hyper-activation is responsible for the impairment of autophagy resulting in reduced clearance of toxic aggregates, that is, Aβ, hyper-phosphorylated tau and oxidized proteins, which accumulates within the brain [12,23,36,40,41,42]. Moreover, we noticed that increased oxidative stress levels and protein damage were associated with dysfunctional BVR-A [12,19,23,26,27,28].

**Figure 6:**
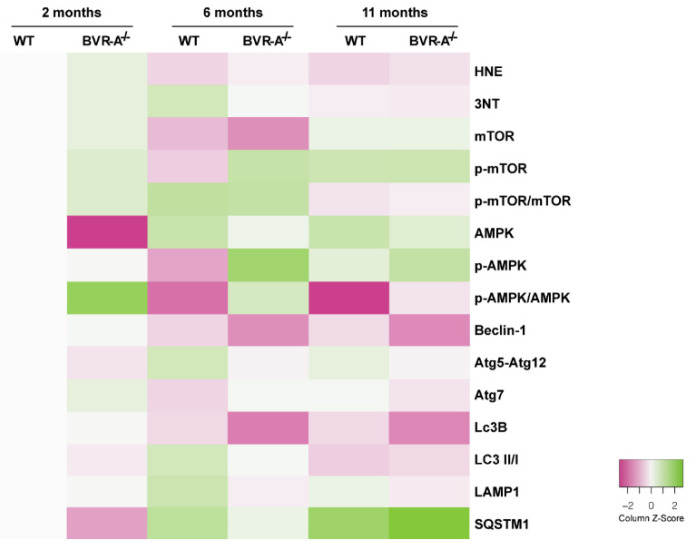
Heat-map showing changes for the proteins analyzed in the study. The heatmap shows a distribution pattern for all the proteins analyzed in the study across the groups of comparison, based on relative abundance (column z-score). The gray square indicates WT mice at 2 months of age set as 100% and used as normalization group. We set Pink/White/Green for our color scheme. Pink represents the lower values, white the middle and green the highest values.

In the present study, we provide further evidence on the molecular mechanisms that link alternations to BVR-A and oxidative protein damage in the brain by focusing on the role of mTOR/autophagy axis. Indeed, our results suggest that loss of BVR-A promotes the hyper-activation of mTOR, which triggers the reduction of autophagy, thus contributing to the accumulation of oxidized proteins in the brain. Such aberrant regulation of protein homeostasis is evident early in the juvenile period (2 months) and persist both in the adult (6 months) and in aged (11 months) BVR-A^−/−^ mice (Figure 6). Intriguingly, we identified the impairment of AMPK as possible cause of mTOR-hyper-activation, thus proposing the existence of a regulatory axis formed by BVR-A/AMPK/mTOR.

Elevation of reactive oxygen species (ROS) and/or reactive nitrogen species (RNS), promotes the formation of toxic protein adducts (i.e., HNE or peroxynitrite (ONOO^−^)). As consequence of increased oxidative and nitrosative damage, protein misfolding, inactivation and aggregation occurs [57]. Neurons respond to the accumulation of oxidatively modified proteins by inducing protein quality control systems, such as ubiquitin proteasome systems (UPS) and autophagy, thereby preserving protein homeostasis. The failure of protein clearance systems, as observed in several neurological disorders, favors the buildup of neurotoxic aggregates and leads to neuronal dysfunctions and deaths [45]. In particular, learning and memory deficits, decreased higher executive function and diminished reasoning ability largely originate from synaptic dysfunction involving oxidatively altered synaptic proteomes [58]. 

Here, we show that BVR-A^−/−^ mice are characterized by an early accumulation of oxidatively-damaged proteins within the brain. This observation is in agreement with the known role for BVR-A as antioxidant protein, mainly due to the production of bilirubin [2]. Indeed, BVR^−/−^ primary neurons, isolated from the knock-out model used in the current study, are hyper-sensitive to superoxide anion (O_2_^•−^) and they cannot detoxify ROS as efficiently as WT. In addition, BVR^−/−^ neurons exhibit increased lipid peroxidation than WT neurons suggesting that endogenous bilirubin protects neurons from excitotoxic oxidative stress [20]. Our data concerning the increase of protein-bound HNE and 3-NT levels agree with that paradigm, keeping in mind that peroxynitrite originates from the reaction between O_2_^•−^ and nitric oxide (NO), while HNE is a lipid peroxidation product [59]. Similarly, Chen et al. reported increased oxidative stress levels in plasma isolated from BVR-A^−/−^ mice independently generated by their group [5]. Furthermore, most studies focused on the possibility that bilirubin and biliverdin act primarily as antioxidants and may reduce oxidative stress damage by suppressing intracellular ROS/RNS [60,61,62]. Results from the current study also strengthen our previous findings showing a close association between impairment of BVR-A and increased oxidative stress levels in the brain of AD and MCI [26,27] as well as DS subjects [28]. In particular, we have demonstrated that BVR-A is associated with an increase in levels of oxidatively damaged proteins and that BVR-A is itself a target of nitrosative modifications (3-NT), that impair its activity and reduce its neuroprotective effects [9,12,19,21,23,24,25,26,27,28].

To note, increased oxidative stress levels elicited by a persistent reduction of BVR-A could be responsible for the impairment of the inducible form of HO, that is, HO-1. Indeed, under conditions of oxidative and nitrosative stress brain reacts by up-regulating genes involved in cell stress response processes to limit neuronal damage [63,64]. HO-1 is among the first proteins induced to elicit an antioxidant response mainly by favoring the degradation of pro-oxidant heme [65,66,67]. Similar to BVR-A, we observed that HO-1, is target of oxidative modifications (mainly HNE) in MCI and AD brain [68]. This event would contribute to further exacerbate the lack of neuroprotective effects normally associated with the up-regulation of the HO-1/BVR-A system in the brain [2,68,69]. Although the role of HO-1 has not been investigated in the current paper, we believe that it will be of great interest to unravel in future studies the effects produced by the lack of BVR-A on HO-1. Particularly, because BVR-A has been shown to function also as transcription factor for HO-1 [6,70]

Besides the antioxidant role for BVR-A, a striking finding of our study concerns the molecular link between BVR-A and mTOR and the potential regulatory role for BVR-A towards the mTOR/autophagy pathway. Our results demonstrate that mTOR becomes hyper-active in mice lacking BVR-A, as result of mTOR increased activation process (p-mTOR^Ser2448^/mTOR), particularly at 2 and 6 months (Figure 2D.1). Instead, higher p-mTOR^Ser2448^ levels in 11 months old animals result from increased mTOR protein levels. Notwithstanding, both cases end up with mTOR hyper-activation supporting the notion that BVR-A could regulate mTOR, as it does with other proteins [1]. Interestingly, in a previous report, Bisht and colleagues showed that high doses of biliverdin (the substrate of BVR-A) promote the activation of the mTOR pathway, while rapamycin (a well-known mTOR inhibitor) prevents biliverdin-mediated effects [71]. Since biliverdin is not known to regulate mTOR catalytic domain or expression levels but it is known to inhibit BVR-A expression at high concentrations [72], its effect on mTOR activation could be mediated by the down-regulation of BVR-A protein levels. This hypothesis is in agreement with studies from our group showing that reduced BVR-A levels or dysfunctional BVR-A are both events associated with the hyper-activation of mTOR in the brain [12,19,23]. In addition, we previously showed that increased oxidative/nitrosative stress levels, mediated by administration of peroxynitrate or H_2_O_2_ to neuroblastoma cells, lead to reduced BVR-A protein levels along with mTOR hyper-activation [12,19], as observed in BVR-A^−/−^ mice.

It is unlikely that BVR-A regulates mTOR phosphorylation directly since it is not a phosphatase. However, BVR-A contains several sequence motifs within its structure that allow BVR-A to interact and to regulate multiple proteins including (i) members of the protein kinase C (PKC) family, (ii) members of the mitogen-activated proteins kinase (MAPK) family and (iii) the insulin receptor substrate-1 (IRS1) among the others (reviewed in Reference [1]). Furthermore, we identified a scaffold role for BVR-A in favoring the Akt-mediated inhibition of GSK3β [21] or in inhibiting the casein kinase-1 (CK1)-mediated phosphorylation of beta β-site APP cleaving enzyme 1 (BACE 1) [23], which are all mechanisms involved in neurodegeneration.

To further unravel whether the hyperactivation of mTOR observed in BVR-A^−/−^ mice results from defects in regulatory mechanisms normally deputed to maintain mTOR activation within a physiological range, we focused on AMPK. AMPK is considered a key cellular energy sensor and it has a crucial role in the control of several processes ranging from lipids biosynthesis and catabolism to glucose uptake and antioxidant defence [73]. AMPK is dysregulated in major metabolic disorders such as diabetes, obesity, as well as in neurodegenerative diseases [74,75].

Under condition of energy depletion, AMPK directly senses increases in AMP:ATP and ADP:ATP ratios, thus promoting the inhibition of mTORC1 complex to inhibit protein synthesis and cell cycle progression, controlling cell size and preventing apoptosis, downstream of mTORC1 inhibition, at times of energy crisis [76]. Moreover, AMPK is activated in response to nitrosative stress and that occurs independently of AMP/ATP levels [77]. Conversely, reduced AMPK activation leads to mTOR hyper-activation [77,78].

Remarkably, we observed that AMPK protein expression is almost null in BVR-A^−/−^ mice at 2 months (Figure 5B.1) and we cannot exclude that a BVR-A-mediated transcriptional control of AMPK exists. However, we believe that reduced AMPK and pAMPK^Thr172^ levels greatly contribute to the marked hyper-activation of mTOR observed in young BVR-A^−/−^ mice at 2 months, in agreement with the regulatory role for AMPK [76,77,78]. Intriguingly, an increase of both AMPK protein and pAMPK^Thr172^ levels in adult and aged BVR-A^−/−^ mice, was observed. Increased pAMPKThr^172^ levels seem to result from increased AMPK protein expression, considering that the activation process (indexed as pAMPK^Thr172^/AMPK ratio) reduces at 6 and 11 months with respect to 2 months (Figure 5D.2). Despite of that, mTOR hyper-activation persists in both adult and aged BVR-A^−/−^ mice. Hence, our hypothesis is that the marked hyper-activation of mTOR observed at 2 months, promotes molecular alterations that cannot be recovered in adult and aged mice. In other words, even if AMPK and pAMPK^Thr172^ levels increase, they are not sufficient to rescue mTOR physiological activation. This is strengthened by the evidence that an increase of AMPK and pAMPK^Thr172^ with age occurs also in WT mice but is not associated with mTOR hyper-activation, suggesting that the AMPK/mTOR mutual regulation is not disrupted. In agreement with that, we did not observe a reduction of autophagy-related protein with age in WT mice.

As expected, the aberrant activation of mTOR is then followed by the impairment of autophagy pathway that results dysfunctional at multiple steps. Indeed, both autophagosome formation and maturation processes are impaired in BVR-A^−/−^ compared to WT mice at 2 months of age as indexed by the early reduction of Beclin-1, LC3β, LC3II/I ratio and Atg5–Atg12 complex levels (Figure 3B.1,C.1 and Figure 4B.1,C.1). Similar alterations, along with reduced Atg7 protein levels, can be observed also in aged mice (11 months).

Surprisingly, no differences are observed between BVR-A^−/−^ and WT mice at 6 months. While this observation, if considered alone, seems to not support the hypothesis that loss of BVR-A impairs autophagy, we believe this was not the case. Indeed, if we take into consideration changes occurring with age, it appears evident that the path of variation for each protein is different between BVR-A^−/−^ and WT mice, thus underlying a dysregulation of the process in mice lacking BVR-A. We acknowledge that data collected in 6-months old BVR-A^−/−^ mice could suggest a compensatory effect aimed to restore autophagy in these animals. However, data collected at 11 months demonstrate that mice lacking BVR-A are characterized by reduced levels of autophagy-related proteins with respect to WT mice, implying that in the absence of BVR-A brain cells fail to rescue the correct activation of autophagy. Hence, reduced autophagy together with reduced BVR-A antioxidants effects would contribute to the accumulation of oxidatively-damaged proteins in the brain of BVR-A^−/−^ mice.

Within the autophagic pathway, the analysis of p62/SQSTM1 protein levels also appear to be of interest. Ubiquitin plays a critical role in protein degradation, as it marks proteins for proteolytic and autophagic degradation and is found in abundance in inclusion bodies [79]. p62/SQSTM1 has a ubiquitin-binding domain, which can associate with ubiquitin-modified proteins and shuttle them to the autophagosome for degradation [80]. Interestingly, either a reduction [80] or an accumulation [81] of p62/SQSTM1 leads to impaired degradation of toxic substrates. Depletion of p62/SQSTM1 levels retards the turnover or degradation of polyubiquitinated proteins [80], whereas increased p62/SQSTM1 levels are evident when a block of the degradative systems occur [81]. Indeed, autophagy is responsible for the degradation of p62; therefore, impairment of autophagy is usually accompanied by massive accumulation of p62 followed by formation of aggregate structures positive for p62 and ubiquitin [82]. We found that levels of p62/SQSTM1 are consistently reduced in BVR-A^−/−^ compared with WT mice at 2 months (Figure 4E.1), thus suggesting a dysfunction of the initial autophagic stage as effect of mTOR hyperactivation. Then we observed a significant accumulation of p62/SQSTM1 at 11 months (Figure 4E.1) that implies an increase of autophagosome formation and maturation with age (as described in Figure 3B.2–D.2 and Figure 4C.2–E.2 but an impairment of cargo degradation step. Alterations of p62/SQSTM1 in BVR-A^−/−^ mice thus agree with the increased 3NT and HNE levels observed in the brain. As previously reported, loss of p62/SQSTM1 leads to increased oxidative stress, possibly eliciting a stress-induced senescence response in astrocytes [83], which can also contribute to the progression of AD and other neurodegenerative diseases [84,85]. Similar to AMPK, the fact that p62/SQSTM1 levels are strongly reduced in BVR-A^−/−^ mice at 2 months spurs the necessity to understand whether BVR-A could regulate in some way the expression of p62/SQSTM1, either directly or indirectly. This aspect is out of the scope of this paper and experiments are ongoing in our lab to clarify a potential mechanism on SQSTM-1 regulation.

## 5. Conclusions

**Figure 7:**
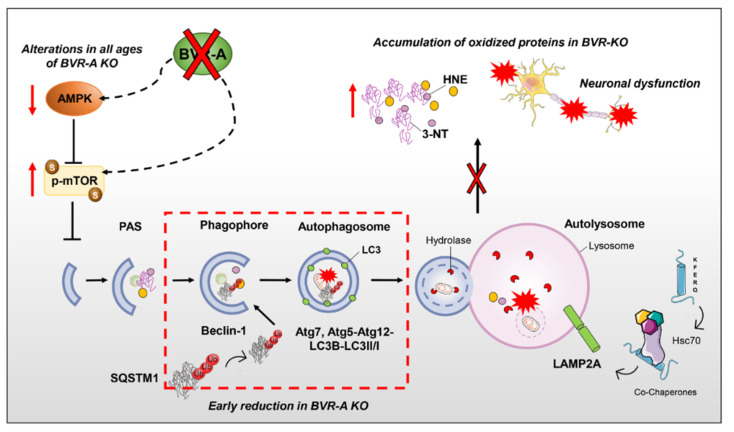
Schematic representation of the autophagosome nucleation/maturation process with highlighted the proposed role for BVR-A in regulating the AMPK/mTOR axis. Under pathological conditions, the hyper-activation of mTOR, mediated by the phosphorylation of serine 2448, is responsible for autophagy inhibition. mTOR activation is maintained within a physiological range by AMPK, that during conditions of reduced energy supply is known to inhibit mTOR, thus favouring the autophagy process. BVR-A deficiency is associated with an early mTOR hyper-activation along with a drastic impairment of AMPK, suggesting the presence of a regulatory axis formed by BVR-A/AMPK/mTOR. In the proposed scenario, we highlight alterations at different steps of the autophagy machinery. In details, autophagosome formation, elongation process and autolysosomal degradation are impaired as indexed by the reduction of Beclin-1, LC3β, LC3II/I ratio Atg5–Atg12 complex and Atg7 levels. Furthermore, alterations of SQSTM1 (sequestosome-1) levels also suggest an early impairment of cargo’s transport into the autophagosome, affecting the clearance of poly-ubiquitinated proteins. As results of impaired autophagy in the brain, an increased accumulation of oxidatively-damaged proteins (HNE and 3-NT) in the brain of BVR-A^−/−^ mice occurs. The failure of autophagic flux further exacerbates the build-up of neurotoxic aggregates, triggering a vicious cycle that gradually results in neuronal dysfunction. Red dashed lines underline early altered steps of autophagy in BVR-A^−/−^ mice.

In conclusion, our study reveals that the loss of BVR-A is a key event promoting mTOR hyper-activation and the consequent impairment of autophagy (Figure 7). Because defects in the autophagy-mediated clearance of toxic aggregates in the brain are associated with the progression of neurodegenerative disorders, that is, MCI and AD, we believe our results would offer a novel molecular target to take into consideration for future studies. Hereafter, our data open the possibility that by modulating BVR-A, it would be possible to improve vesicular trafficking and consequently restore normal autophagic activity in the brain.

## Figures and Tables

**Table 1:** 2-way ANOVA analysis of western blot data for the identification of the influence of age and genotype on the differences observed between WT and BVR-A^−/−^ mice at 2, 6 and 11 months of age.

Target of Analysis	2-WAY ANOVA					
	Age		Genotype (WT-BVR-A^−/−^)		Interaction	
	F (DFn, DFd)	*p*	F (DFn, DFd)	*p*	F (DFn, DFd)	*p*
**HNE**	F (2, 18) = 5.18	*p* < 0.05	n.s.	n.s	n.s.	n.s.
**3-NT**	F (2, 18) = 28,70	*p* < 0,0001	n.s.	n.s.	F (2, 18) = 5,141	*p* < 0,05
**p-mTOR**	F (2, 18) = 10.53	*p* < 0.001	F (1, 18) = 40.78	*p* < 0.0001	n.s.	n.s.
**mTOR**	F (2, 18) = 45.89	*p* < 0.0001	F (1, 18) = 5.33	*p* < 0.05	n.s.	n.s.
**p-mTOR/mTOR**	F (2, 18) = 6.43	*p* < 0.01	F (1, 18) = 16.40	*p* < 0.001	F (2, 18) = 4.02	*p* < 0.05
**Beclin-1**	F (2, 18) = 5.08	*p* < 0.05	F (1, 18) = 7.52	*p* < 0.05	F (2, 18) = 5.59	*p* < 0.05
**Atg12-Atg5**	F (2, 18) = 20.30	*p* < 0.0001	F (1, 18) = 9.27	*p* < 0.01	n.s.	n.s.
**Atg7**	F (2, 18) = 8.26	*p* < 0.01	n.s.	n.s.	F (2, 18) = 7.34	*p* < 0.01
**LC3** **β**	n.s.	n.s.	n.s.	n.s.	n.s.	n.s.
**LC3 II/I**	F (2, 18) = 49.28	*p* < 0.0001	F (1, 18) = 16.58	*p* < 0.001	F (2, 18) = 15.50	*p* < 0.0001
**SQSTM1**	F (2, 18) = 25.27	*p* < 0.0001	n.s.	n.s.	F (2, 18) = 5.02	*p* < 0.05
**LAMP1**	F (2, 18) = 5.54	*p* < 0.05	n.s.	n.s.	n.s.	n.s.
**AMPK**	F (2, 18) = 18.45	*p* < 0.0001	n.s.	n.s.	n.s.	n.s.
**p-AMPK**	F (2, 18) = 7.42	*p* < 0.01	F (1, 18) = 11.55	*p* < 0.01	F (2, 18) = 5.74	*p* < 0.05
**p-AMPK/AMPK**	F (2, 18) = 19.88	*p* < 0.0001	F (1, 18) = 36.12	*p* < 0.0001	F (2, 18) = 17.40	*p* < 0.0001

**Table 2:** Correlation analysis between p-mTOR (Ser2448) and proteins involved in autophagy pathway. Significant values are in bold.

Target of Analysis	*WT*		*BVR-A* ^−/−^	
	*r*	*p*	*r*	*p*
**p-AMPK**	0.445	n.s.	0.086	n.s.
**AMPK**	**0.584**	**<0.05**	0.468	n.s.
**p-AMPK/AMPK**	0.114	n.s.	−0.339	n.s.
**Beclin-1**	**0.688**	**<0.01**	−0.240	n.s.
**Lamp1**	**0.622**	**<0.05**	0.476	n.s.
**SQSTM-1**	**0.579**	**<0.05**	0.480	n.s.
**Atg5-12**	**0.676**	**<0.05**	0.467	n.s.
**Atg7**	**0.653**	**<0.05**	0.307	n.s.
**Lc3** **β**	**0.725**	**<0.01**	0.420	n.s.
**LC3 II/I**	−0.231	n.s.	0.021	n.s.

## Abbreviations

AD	Alzheimer’s disease
ATG	autophagy-related proteins
AMPK	5’ adenosine monophosphate-activated protein kinase
BIR	brain insulin resistance
IRS-1	insulin receptor substrate-1
BVR	Biliverdin reductase
HO	heme oxygenase
IGF-1	insulin-like growth factor-1
LAMP	lysosome-associated membrane protein
LC3	microtubule-associated protein 1A/1B-light chain 3
KO	knock-out
MAPK	mitogen-activated protein kinase
MCI	mild cognitive impairment
mTORC1	mammalian target of rapamycin complex 1
mTOR	mammalian target of rapamycin
OS	oxidative stress
PBMC	peripheral blood mononuclear cells
PI3K	phosphatidylinositol-3-kinase
SQSTM1	sequestosome-1
3-NT	3-nitro-tyrosine
HNE	4-hydroxy-2-nonenals
TLR4	Toll like receptor 4
